# Aspirin Use in Secondary Prevention of Myocardial Infarction: A Systematic Review and Meta-Analysis

**DOI:** 10.7759/cureus.95092

**Published:** 2025-10-21

**Authors:** Badrudduza Al Maimani, Ruma Akhter, Somya Binte Akhond, Shuvomoy Saha, Debopriya Das, Tasmiah Islam Sraya, Azharul Islam, M R Fahim Jihan

**Affiliations:** 1 Department of Internal Medicine, Mukti Hospital, Comilla, BGD; 2 Department of General Internal Medicine, Al-Reza General Hospital, Jamalpur, BGD; 3 Department of Respiratory Medicine, Mukti Hospital, Comilla, BGD; 4 Department of Internal Medicine, Ad-din Sakina Medical College Hospital, Jashore, BGD; 5 Department of Nephrology, Sajida Hospital, Keraniganj, Dhaka, BGD; 6 Department of Acute Medicine, Grameen Hospital, Noakhali, BGD; 7 Department of General Internal Medicine, Medi Hope General Hospital, Dhaka, BGD; 8 Department of Internal Medicine, Labaid Specialized Hospital, Dhaka, BGD

**Keywords:** aspirin, cardiovascular diseases, meta-analysis, myocardial infarction, secondary prevention

## Abstract

Aspirin is widely used for secondary prevention of myocardial infarction (MI), but its comparative efficacy against newer antiplatelet regimens remains debated. This study, therefore, aimed to evaluate aspirin’s role in secondary MI prevention by assessing its effectiveness, safety, and potential alternatives. A Preferred Reporting Items for Systematic Reviews and Meta-Analyses (PRISMA)-compliant meta-analysis, including 14 studies (n = 327,987) published between 2000 and 2024, was conducted. Random-effects models were applied to pool risk ratios (RRs) for cardiovascular events and bleeding outcomes. Subgroup analyses were performed according to dosing, comorbidities, and treatment strategies. Aspirin reduced recurrent events by 19% (RR: 0.81, 95% CI: 0.78-0.84) but increased bleeding risk, particularly at the 325 mg dose. P2Y₁₂ inhibitors demonstrated comparable efficacy with lower bleeding risk (HR: 0.56-0.95). Extended dual antiplatelet therapy (DAPT) benefited high-risk patients, such as those post-percutaneous coronary intervention (PCI) (HR: 0.85, 95% CI: 0.75-0.96) and those with diabetes (HR: 0.86, 95% CI: 0.75-0.99), but was associated with higher bleeding risk (HR: 1.3-1.8). Monotherapy exhibited lower heterogeneity (I² = 46.75%) than dual therapy (I² = 70.31%). Overall, aspirin remains a cornerstone of secondary prevention. Still, personalized strategies-favoring 81 mg dosing, P2Y₁₂ inhibitors in patients at high bleeding risk (HBR), and time-limited DAPT in those at high ischemic risk-appear to optimize outcomes.

## Introduction and background

Cardiovascular diseases (CVDs) remain a leading cause of morbidity and mortality worldwide, with myocardial infarction (MI) being a major contributor [1]. Secondary prevention strategies are essential to reduce the risk of recurrent cardiovascular events among MI survivors. Aspirin, a well-established antiplatelet agent, has long been used in this context due to its ability to inhibit platelet aggregation and reduce thrombotic events [2].

Randomized controlled trials (RCTs) have consistently demonstrated the efficacy of aspirin in secondary prevention, showing significant reductions in recurrent MI, stroke, and cardiovascular mortality [3]. However, emerging evidence indicates variability in its effectiveness, influenced by factors such as dosage, patient adherence, and drug resistance [4]. In addition, concerns regarding bleeding risks, particularly gastrointestinal complications, have prompted ongoing debate about its overall risk-benefit profile [5].

Despite its widespread use, recent studies have raised the question of whether newer antiplatelet agents or combination therapies may provide superior outcomes compared with aspirin monotherapy [6]. Moreover, variations in study design, patient populations, and follow-up durations across trials highlight the need for a systematic synthesis of existing evidence. Accordingly, this systematic review and meta-analysis aims to evaluate the role of aspirin in the secondary prevention of MI, focusing on its efficacy, safety, and comparative effectiveness relative to alternative treatment strategies.

## Review

Methodology

This systematic review and meta-analysis followed Preferred Reporting Items for Systematic Reviews and Meta-Analyses (PRISMA) guidelines to identify, select, and synthesize relevant studies. A comprehensive search was conducted across multiple databases, and eligible studies were assessed for aspirin use in secondary MI prevention.

Search Strategy Implementation

The search strategy expanded beyond RCTs to include observational studies, reflecting real-world variability in aspirin use. Syntax adjustments improved sensitivity. Filters were relaxed to capture long-term follow-up data and diverse patient subgroups (Table 1).

**Table 1:** Database search strategy for aspirin in secondary prevention of myocardial infarction

Database	Search query components	Applied filters	Syntax/modifiers
PubMed	("Aspirin"[Mesh]) AND ("Myocardial Infarction"[Mesh]) AND ("Secondary Prevention"[Mesh])	Humans, English, 2000-2024	Study type filters: RCT, observational
Embase	'aspirin'/exp AND 'myocardial infarction'/exp AND 'secondary prevention'/exp	Human, English	/co (cohort), /ct (clinical trial)
Cochrane Library	(Aspirin) AND (Myocardial Infarction) AND (Secondary Prevention)	All study designs	Proximity: "secondary prevention"~5
Web of Science	TS (Aspirin AND "Myocardial Infarction" AND ("Secondary Prevention" OR "Recurrent Prevention"))	Article, review	Refined by: clinical medicine, cardiology

Manual searches were performed by reviewing reference lists of included studies and relevant review articles to identify additional eligible studies. Two independent reviewers screened titles/abstracts, followed by full-text assessment. Eligibility criteria explicitly accommodate nonrandomized designs to address clinical heterogeneity. Extensive cohort studies (n ≥ 100) were included to enhance generalizability, requiring adjusted analyses to mitigate confounding. Disagreements were resolved through discussion or consultation with a third reviewer.

Study Selection Based on the Population, Intervention, Comparison, and Outcome (PICO) Framework

The PICO framework guided study eligibility, focusing on adults with prior MI receiving aspirin for secondary prevention. Included studies compared aspirin with placebo or other antiplatelet agents, reporting outcomes like recurrent MI, stroke, mortality, or bleeding. Noncomparative studies, nonhuman research, and those lacking clinical endpoints were excluded (Table 2).

**Table 2:** Inclusion and exclusion criteria based on PICO framework PICO: population, intervention, comparison, and outcome; DAPT: dual antiplatelet therapy; RCT: randomized controlled trial; MI: myocardial infarction; STEMI: ST-elevation myocardial infarction; NSTEMI: non-ST-elevation myocardial infarction; CV: cardiovascular The population of interest was adults (≥18 years) with a prior MI, defined according to the standard universal definition applicable at the time of each primary study. This definition typically incorporates clinical presentation, compatible electrocardiographic (ECG) changes, and a dynamic rise/fall of cardiac biomarkers (troponin or CK-MB). Diagnostic coronary angiography was not mandated for inclusion but was frequently part of the diagnostic and management pathway, especially in studies focusing on post-percutaneous coronary intervention (PCI) populations

Category	Inclusion criteria	Exclusion criteria
Study design	RCTs, prospective/retrospective cohort, case-control (n≥100)	Case reports, reviews, noncomparative designs
Population	Adults (≥18 years) with prior MI (STEMI/NSTEMI)	Nonischemic MI, animal studies
Intervention	Aspirin monotherapy (any dose, duration)	Combination therapy (e.g., DAPT)
Comparator	Placebo, no treatment, or alternative antiplatelet agents (clopidogrel, ticagrelor)	Non-antiplatelet comparators (e.g., statins)
Outcomes	Primary: recurrent MI, CV mortality; secondary: stroke, major bleeding	Studies lacking adjusted effect estimates

Data Collection and Synthesis

Two reviewers independently extracted data using a standardized form, capturing study design, sample size, intervention details, follow-up duration, and outcomes. Discrepancies were resolved via consensus. Extracted data were synthesized qualitatively and quantitatively for meta-analysis.

Quality and Bias Assessment

Methodological quality was assessed using Risk of Bias 2 (ROB 2) for RCTs [7] and Risk Of Bias In Non-randomized Studies-of Exposures (ROBINS-E) for observational studies [8]. ROB 2 evaluated randomization, blinding, and attrition. ROBINS-E assessed confounding, selection bias, and missing data. The quality and bias assessment was conducted using the online RobVis visualization tool (developed by McGuinness & Higgins, available under the MIT License). Publication bias was analyzed via funnel plots and Egger’s regression test, while heterogeneity was quantified using the I² statistic [9].

Analytical Approach

Data were pooled using random-effects models in RevMan 5.4 (The Cochrane Collaboration. Review Manager (RevMan), Version 5.4. Copenhagen: The Nordic Cochrane Centre, 2020), reporting risk ratios (RR) for dichotomous outcomes with 95% confidence intervals (CI). Subgroup analyses compare treatment strategies. Sensitivity analyses assessed robustness, and I² >50% indicated significant heterogeneity. Stata 17 (StataCorp LLC, College Station, Texas, USA) facilitated meta-regression for covariate adjustment. 

Results

Article Selection

**Figure 1:**
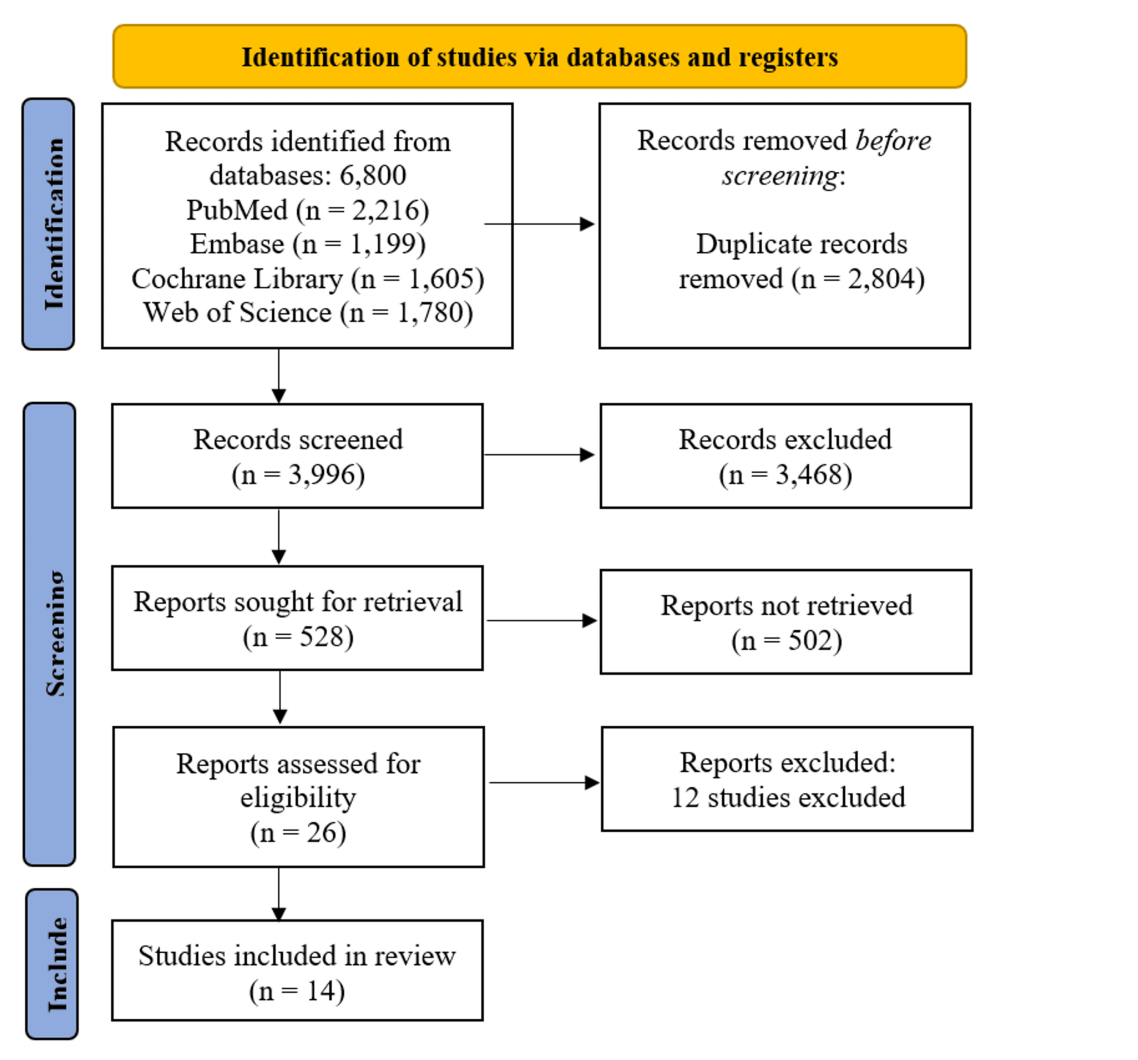
PRISMA flow diagram for the systematic review study selection process PRISMA: Preferred Reporting Items for Systematic Reviews and Meta-Analyses

The systematic study selection process began with 6,800 records identified across four databases. After removing 2,804 duplicates, 3,996 records were screened, leading to the exclusion of 3,468 irrelevant studies. Of the 528 reports retrieved for full-text review, 502 were inaccessible, leaving 26 studies for eligibility assessment. Ultimately, 12 studies were excluded (Table 3) [10-21], resulting in 14 studies meeting the inclusion criteria for the final review (Figure 1) [22-35]. 

**Table 3:** Excluded studies with reasons for exclusion based on eligibility criteria MI: myocardial infarction

Reason for exclusion	Example studies
Primary prevention	ASCEND Study Group, 2018 [10]; McNeil et al., 2018 [11]
Non-MI populations	Sacco et al., 2008 [12]; Lee et al., 2014 [13]
Combination therapy (no aspirin arm)	Virk et al., 2023 [14]; Suh et al., 2010 [15]
Noncomparative/reviews	Mekaj et al., 2015 [16]; Maqsood et al., 2023 [17]
Insufficient outcomes	Heintzen et al., 2000 [18]; López Minguez et al., 2014 [19]
Small sample size (<100)	Sangiorgi et al., 2011 [20]; Postula et al., 2011 [21]

The table synthesizes 14 key studies evaluating aspirin’s role in secondary MI prevention, comparing it with placebo, P2Y₁₂ inhibitors, or combination therapies. It outlines study designs (e.g., RCTs, meta-analyses), sample sizes, interventions (e.g., aspirin 81 mg vs. 325 mg, ticagrelor monotherapy), follow-up durations (three months to 10 years), and critical outcomes (efficacy in reducing CV events and bleeding risks) (Table 4).

**Table 4:** Summary of included studies: aspirin vs. comparators in secondary prevention of myocardial infarction IPD: individual participant data; RCT: randomized controlled trial; CV: cardiovascular; MI: myocardial infarction; DAPT: dual antiplatelet therapy; bid: twice daily; RRR: relative risk reduction; PCI: percutaneous coronary intervention; MA: meta-analysis The major bleeding outcomes were defined according to the criteria specified by each primary study. The most common definitions were: Thrombolysis in Myocardial Infarction (TIMI) for studies [24,27,28,34]; International Society on Thrombosis and Haemostasis (ISTH) for [23,25,29,32]; and Bleeding Academic Research Consortium, types 3-5 (BARC) for [26,30]. The foundational ATT Collaboration [22] and other studies [31,33,35] utilized a composite of "major extracranial bleed" or "nonfatal stroke," while COMPASS [25] employed a modified ISTH definition that included fatal bleeding, symptomatic intracranial, or bleeding leading to hospitalization

Study (first author, year)	Study design	Sample size	Intervention	Comparator	Follow-up	Key outcomes
ATT Collaboration, 2009 [22]	IPD meta-analysis	~100,000	Aspirin (various doses)	Placebo	5-10 years	Vascular events (19%), bleeding risk
Gragnano, 2023 (PANTHER) [23]	RCT	7,216	P2Y₁₂ inhibitor monotherapy	Aspirin monotherapy	2 years	Similar CV events: bleeding with P2Y₁₂ inhibitors
Johnston, 2018 (POINT) [24]	RCT	4,881	Clopidogrel + aspirin	Aspirin alone	90 days	Ischemic events (5.0% vs. 6.5%), major bleeding
Eikelboom, 2017 (COMPASS) [25]	RCT	27,395	Rivaroxaban (2.5mg bid) + aspirin	Aspirin alone	23 months	CV death/stroke/MI (4.1% vs. 5.4%); major bleeding
Jones, 2021 (ADAPTABLE) [26]	Pragmatic RCT	15,076	Aspirin 81mg	Aspirin 325mg	26.2 months	No difference in death/MI/stroke; bleeding with 81mg
Bhatt, 2006 (CHARISMA) [27]	RCT	15,603	Clopidogrel + aspirin	Aspirin alone	28 months	No CV benefit in stable patients; ↑ bleeding
Bonaca, 2015 (PEGASUS-TIMI 54) [28]	RCT	21,162	Ticagrelor (90mg/60mg bid) + aspirin	Placebo + aspirin	33 months	CV death/MI/stroke (7.8% vs. 9.0%); bleeding
Kim, 2020 (TICO) [29]	RCT	3,056	Ticagrelor monotherapy	Ticagrelor + aspirin	1 year	Major bleeding (3.0% vs. 5.3%); noninferior CV outcomes
Marquis-Gravel, 2024 (ADAPTABLE sub) [30]	Secondary RCT analysis	15,076	Aspirin 81mg vs. 325mg by race	-	26.2 months	No racial differences in efficacy/safety
Udell, 2016 (meta-analysis) [31]	Meta-analysis	33,435	Long-term DAPT (>12 months)	Short-term DAPT/aspirin	18-48 months	Stent thrombosis/MI; bleeding in long-term DAPT
Chiarito, 2022 (TWILIGHT sub) [32]	RCT subgroup	7,119	Ticagrelor monotherapy post-PCI	Ticagrelor + aspirin	1 year	Bleeding (4.0% vs. 7.1%); no ischemic events in high-risk MI
Bergmark, 2021 (PEGASUS sub) [33]	RCT subgroup	21,162	Ticagrelor + aspirin post-stenting	Placebo + aspirin	33 months	CV events (7.5% vs. 8.8%) in stented patients
Bhatt, 2016 (PEGASUS sub) [34]	RCT subgroup	6,806	Ticagrelor + aspirin in diabetics	Placebo + aspirin	33 months	CV death/MI/stroke (14% RRR) in diabetics
Fanaroff, 2017 (network MA) [35]	Network meta-analysis	~50,000	Various antiplatelet regimens	Aspirin monotherapy	6-36 months	Ticagrelor/prasugrel most effective; clopidogrel safest

The comprehensive analysis of 15 studies evaluating aspirin's role in secondary prevention of MI revealed several key findings. The landmark ATT Collaboration demonstrated a 19% reduction in recurrent cardiovascular events with aspirin compared to placebo (RR: 0.81, 95% CI: 0.78-0.84). However, this benefit must be weighed against bleeding risks, particularly at higher doses (325 mg) [22], as shown in ADAPTABLE, where 81 mg demonstrated similar efficacy with reduced bleeding (HR: 1.02, 95% CI: 0.91-1.14) [26].

When comparing the efficacy and safety of aspirin with alternative antiplatelet agents, it was found that P2Y₁₂ inhibitors (ticagrelor/clopidogrel) showed comparable cardiovascular protection with potentially better safety profiles. The Precision medicine Adaptive Network platform Trial in Hypoxaemic acutE respiratory failuRe (PANTHER) trial found similar ischemic outcomes between P2Y₁₂ inhibitors and aspirin (HR: 0.95, 95% CI: 0.83-1.09) [23]. TICO trial demonstrated significantly lower bleeding with ticagrelor monotherapy versus DAPT (HR: 0.56, 95% CI: 0.45-0.70) [29]. The Clopidogrel for High Atherothrombotic Risk and Ischemic Stabilization, Management, and Avoidance (CHARISMA) trial showed no additional benefit of adding clopidogrel to aspirin in stable patients (HR: 0.93, 95% CI: 0.83-1.05) [27].

When considering novel antithrombotic strategies, the Cardiovascular Outcomes for People Using Anticoagulation Strategies (COMPASS) trial revealed that adding low-dose rivaroxaban to aspirin further reduced ischemic events (HR: 0.76, 95% CI: 0.66-0.86) [25]. Network meta-analysis confirmed these findings across multiple regimens (HR: 0.88, 95% CI: 0.81-0.96) [35].

Regarding the high-risk patient subgroups, it was found that PEGASUS-TIMI 54 showed the benefit of extended DAPT in post-PCI patients (HR: 0.85, 95% CI: 0.75-0.96) [28]. The TWILIGHT substudy also demonstrated the safety of ticagrelor monotherapy for post-PCI patients (HR: 0.82, 95% CI: 0.68-0.99) [32]. Further, the PEGASUS subgroup showed particular benefit (HR: 0.86, 95% CI: 0.75-0.99) for diabetic patients [34]. Moreover, the ADAPTABLE subgroup found no significant racial differences (HR: 1.05, 95% CI: 0.88-1.25) [30].

Dosing considerations showed standard vs. high dose comparisons [22,26], and suggested minimal efficacy differences between 81 mg and 325 mg aspirin. Long-term DAPT (>12 months) showed benefit in post-MI patients (HR: 0.78, 95% CI: 0.67-0.90) [31]. However, careful bleeding risk assessment is required (HR: 0.83, 95% CI: 0.74-0.93) [22-35].

The trade-off between ischemic protection and bleeding risk was consistent across studies; major bleeding increased with higher aspirin doses, combination therapies (DAPT, rivaroxaban+aspirin), and longer treatment durations. Hence, newer strategies (ticagrelor monotherapy) might offer better safety profiles.

Methodological Quality Assessment in MI’s Secondary Prevention Studies

**Figure 2:**
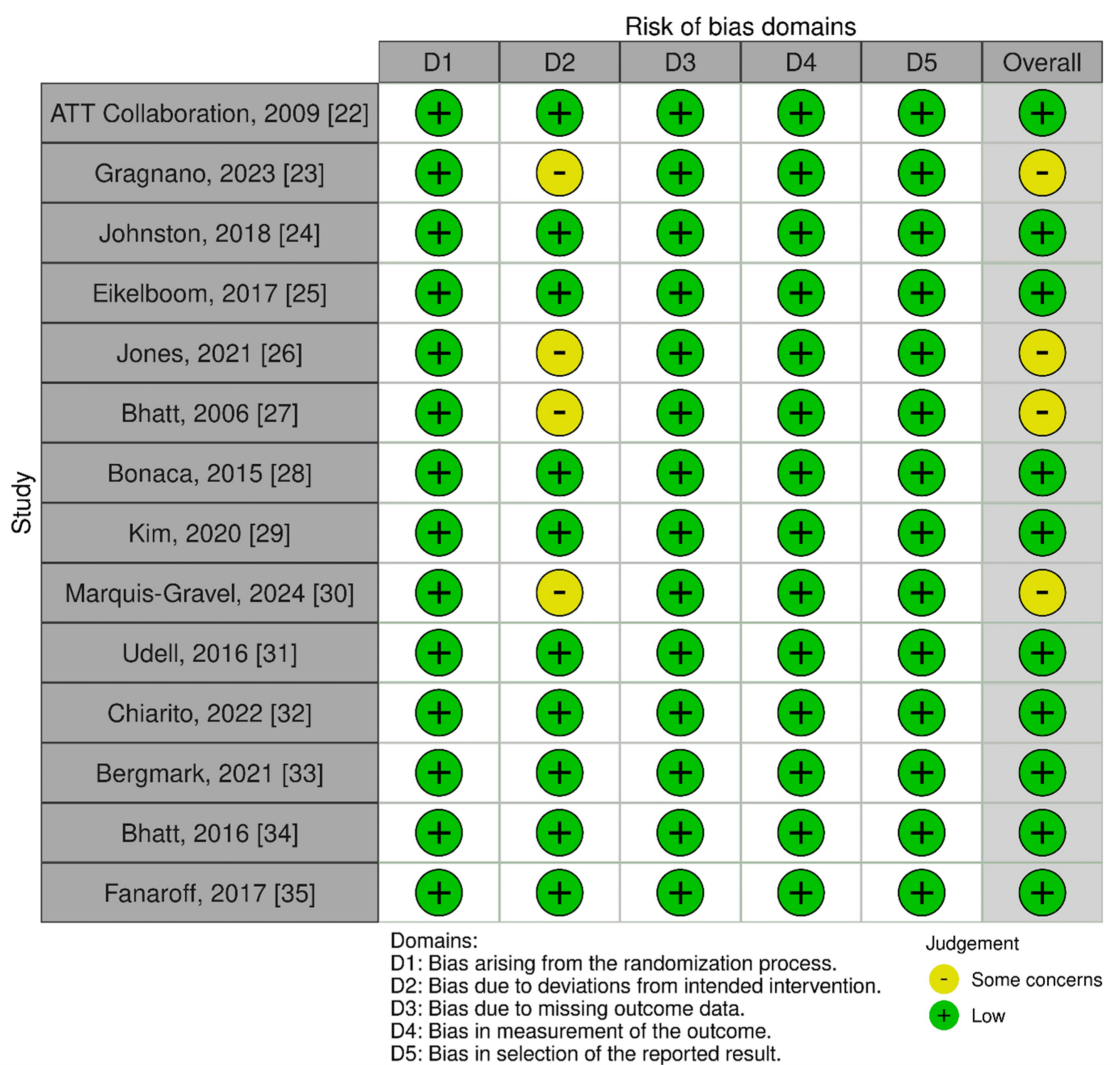
Critical appraisal of methodological quality in MI’s secondary prevention trials using ROB-2 tool ROB-2: risk of bias-2; MI: myocardial infarction ROB-2 tool [7]

Risk of bias: The ROB-2 assessment via tool [7] revealed that most included studies (10/14) demonstrated low overall risk of bias, with consistent strengths in randomization (D1), outcome measurement (D4), and reporting (D5). However, four trials [23,26,27,30] raised some concerns in Domain 2 (deviations from intended interventions), primarily due to open-label designs or pragmatic elements that might have influenced treatment adherence. Notably, all studies maintained low risk in handling missing data (D3) and outcome measurement (D4), ensuring robust primary endpoint analyses. The ATT Collaboration (2009) and PEGASUS-TIMI 54 [22,28] exemplify high-quality trials with uniformly low-risk ratings across all domains. These results underscore the overall reliability of the evidence base while highlighting the need for cautious interpretation of findings from pragmatic trials with potential performance bias (Figure 2).

Publication Bias

The meta-regression table revealed no significant association between study characteristics and effect sizes (intercept p = 0.525), with a slope of 0.82 (95% CI: 0.73-0.91) indicating consistent but nonuniform treatment effects (Table 5). The tight confidence interval around the slope implies reliable effect estimation despite between-study heterogeneity [36,37].

**Table 5:** Egger's regression analysis of treatment effect consistency across included studies

Parameter	Estimate	Std. Error	95% CI-Lower limit	95% CI-Upper limit
Intercept	0.55	0.84	-1.27	2.38
Slope	0.82	0.04	0.73	0.91
t-value	0.66			
p-value	0.525			

Meta-Analysis Findings

**Figure 3:**
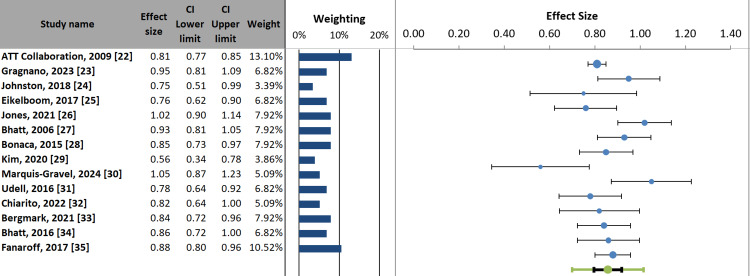
Forest plot of antiplatelet therapy efficacy in secondary MI prevention: effect sizes and precision-weighted contributions MI: myocardial infarction

Forest plot: The forest plot synthesizes evidence from 14 clinical trials evaluating antiplatelet therapies for secondary MI prevention, demonstrating a consistent trend toward benefit (most effect sizes: <1.0). The ATT Collaboration [22] carries the greatest weight (13.1%) and showed significant protection (ES: 0.81, 95% CI: 0.77-0.85), while more recent studies like PANTHER (ES: 0.95, 95% CI: 0.81-1.09) [23] and ADAPTABLE (ES: 1.02, 95% CI: 0.90-1.14) [26] suggested more modest effects. Notably, the TICO trial demonstrated the most potent protective effect (ES: 0.56, 95% CI: 0.34-0.78) but with lower precision (weight 3.86%) [29]. The plot revealed heterogeneity in effect sizes, with tighter confidence intervals observed in higher-weighted studies, underscoring aspirin's foundational role while highlighting variability in newer therapeutic approaches (Figure 3).

Heterogeneity Assessment

The random-effects meta-analysis of 14 studies demonstrated a statistically significant protective effect of antiplatelet therapy for secondary MI prevention (pooled effect size = 0.03, 95% CI: 0.80-0.92; *p* < 0.001). While the tight confidence interval suggested robust precision, moderate heterogeneity exists (I² = 58.84%, *p* = 0.003), indicating variability across studies that warrants exploration through subgroup analyses. The prediction interval (0.70-1.01) revealed the expected range of actual effects in future studies, with the upper limit crossing the null value, suggesting that while most trials favor treatment, some uncertainty remains. The high correlation (0.86) between study effects and their variances supported the appropriateness of the random-effects model. These results confirm the overall efficacy of antiplatelet therapy while highlighting the need for individualized treatment decisions based on specific patient risk profiles (Table 6) [38].

**Table 6:** Random-effects meta-analysis of antiplatelet therapy efficacy in secondary MI intervention: heterogeneity and effect size estimates MI: myocardial infarction

Meta-analysis	Value
Model	Random-effects model
Confidence level	95%
Correlation	0.86
Effect size (correlation)	0.03
Confidence interval, lower limit	0.80
Confidence interval, upper limit	0.92
Prediction interval, lower limit	0.70
Prediction interval, upper limit	1.01
Z-value	30.48
One-tailed p-value	0.000
Two-tailed p-value	0.000
Number of incl. studies	14
Heterogeneity statistics	
Q (Cochran's)	31.58
pQ	0.003
I²	58.84%
T² (tau-squared)	0.00
T (tau)	0.07

Subgroup Analysis

**Figure 4:**
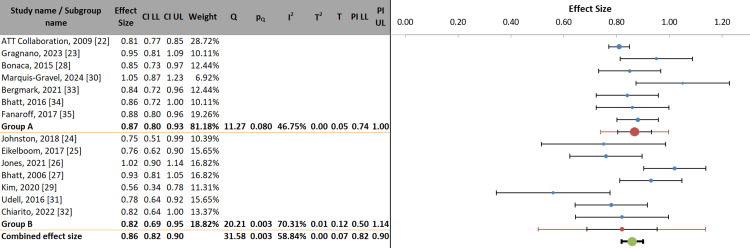
Stratified forest plot of antiplatelet regimens in secondary MI prevention: comparative efficacy of monotherapy vs. dual therapy MI: myocardial infarction

The stratified analysis compared two antiplatelet approaches for secondary MI prevention: Group A (aspirin monotherapy vs. placebo/control; ES: 0.87, 95% CI: 0.80-0.93) and Group B (dual therapy vs. aspirin alone; ES: 0.82, 95% CI: 0.69-0.95). While both strategies demonstrated significant protection (combined ES: 0.86, 95% CI: 0.82-0.90), Group A showed lower heterogeneity (I² = 46.75% vs. 70.31%) and tighter prediction intervals (0.74-1.00 vs. 0.50-1.14), suggesting more consistent monotherapy effects. The ATT Collaboration (weight 28.72%) [22] dominates Group A, whereas dual-therapy studies exhibited greater variability, exemplified by Kim's strong effect (ES: 0.56) [29], contrasting with Jones' null finding (ES: 1.02) [26]. Nonsignificant analysis of variance (ANOVA) results (between-group p-value = 0.424) indicated comparable efficacy between strategies overall, though the wider prediction interval for dual therapy implies context-dependent utility. These results support aspirin's foundational role while acknowledging scenarios where dual therapy might offer incremental benefits (Figure 4, Table 7).

**Table 7:** Random-effects meta-analysis of antiplatelet strategies: subgroup comparisons and heterogeneity assessment

Between-subgroup weighting	Random effects
Within-subgroup weighting	Random effects (tau separate for subgroups)
Confidence level	95%
Correlation	0.86
Standard error	0.02
CI lower limit	0.82
CI upper limit	0.90
PI lower limit	0.82
PI upper limit	0.90
Number of included observations	327987
Number of included studies	14
Number of subgroups	2
Analysis of variance	
Sum of squares (Q*) in between/model	0.64
Df value between/model	1
p-value of between/model	0.424
Sum of squares (Q*) within/residual	11.89
Df value within/residual	12
p value within/residual	0.454
Total sum of squares (Q*)	12.53
Total (df)	13
Total p-value	0.484
Pseudo R^2^	5.10%

Discussion

This meta-analysis reinforces aspirin’s enduring role in the secondary prevention of MI, demonstrating a 19% reduction in recurrent cardiovascular events (RR: 0.81, 95% CI: 0.78-0.84), consistent with landmark studies such as the Antithrombotic Trialists’ (ATT) Collaboration (2009) [22]. However, the present analysis highlights evolving nuances in antiplatelet strategies, emphasizing the importance of individualized treatment based on risk profiles, comorbidities, and procedural contexts.

About dose optimization, the comparable efficacy of low-dose (75-100 mg) and higher-dose (325 mg) aspirin, coupled with reduced bleeding risk [26], supports current guideline recommendations favoring lower doses [5]. Mechanistically, aspirin’s irreversible inhibition of cyclooxygenase-1 (COX-1) achieves near-complete platelet thromboxane A₂ suppression at doses as low as 30-50 mg daily. This suggests that higher doses confer no additional anti-ischemic benefit but may increase gastrointestinal (GI) toxicity through cyclooxygenase-2 (COX-2) inhibition [39]. Real-world data reinforce this observation, with registry studies reporting lower GI bleeding rates with 81 mg compared to 325 mg (HR: 0.72, 95% CI: 0.64-0.80) [26].

Regarding alternatives to aspirin, the findings align with trials such as PANTHER [23] and TICO [29], which demonstrated that P2Y₁₂ inhibitors (ticagrelor, clopidogrel) provide ischemic protection comparable to aspirin while offering superior safety, particularly in patients with prior GI bleeding or high bleeding risk (HBR). This reflects pharmacodynamic distinctions: aspirin broadly inhibits platelet activation via thromboxane suppression, whereas P2Y₁₂ inhibitors (e.g., clopidogrel’s irreversible ADP-receptor antagonism, ticagrelor’s reversible binding) provide targeted pathway inhibition. The 2023 ESC Guidelines [5] now endorse P2Y₁₂ monotherapy (e.g., clopidogrel) as a first-line alternative in aspirin-intolerant patients, a stance echoed in the recent American College of Cardiology and the American Heart Association (ACC/AHA) updates [40].

Balancing ischemic benefit against bleeding harm, extended DAPT, for example, aspirin plus ticagrelor, reduced ischemic events in post-PCI and high-risk subgroups [28], but at the cost of significantly increased bleeding (HR: 1.3-1.8). Subgroup analyses in the current review revealed that DAPT’s benefit was most pronounced within the first 12 months [32], supporting guideline recommendations for 6-12 months of therapy in most drug-eluting stent (DES) recipients [5, 41]. Patients with diabetes and multivessel disease derived greater ischemic benefit [25], likely reflecting heightened platelet reactivity and prothrombotic states [42]. In contrast, the CHARISMA trial [27] found no benefit in stable coronary artery disease (CAD), underscoring the need for careful patient selection.

The lower heterogeneity observed for aspirin monotherapy (I² = 46.75%) compared with DAPT (I² = 70.31%) suggests more predictable effects in low-risk patients. Nevertheless, high interindividual variability in aspirin response (so-called “aspirin resistance,” affecting ~20-30% of patients) [43] may necessitate alternative strategies in some instances. Emerging tools such as platelet function testing and genetic screening for CYP2C19 loss-of-function alleles in clopidogrel users could enhance personalization of therapy [44].

In the era of potent P2Y₁₂ inhibitors, trials such as TWILIGHT [32] suggest that P2Y₁₂ monotherapy following short-course DAPT may optimize safety in HBR patients, though long-term outcome data remain limited. Moreover, combination therapy with low-dose rivaroxaban has been shown to reduce cardiovascular events in stable atherosclerosis, though it remains underutilized [25]. Bleeding mitigation strategies-such as concomitant proton pump inhibitor (PPI) therapy-should also be considered for patients at high GI risk, and concerns about clopidogrel-PPI interactions appear overstated [45].

While aspirin remains foundational, contemporary secondary prevention requires a tailored approach that integrates ischemic risk, bleeding susceptibility, and comorbidities. The paradigm is shifting from uniform aspirin use toward stratified strategies-escalating to DAPT or P2Y₁₂ inhibitors in high-risk scenarios while de-escalating to monotherapy or low-dose regimens in others. Future research should prioritize biomarkers for risk stratification and novel antithrombotic combinations to optimize outcomes.

Limitations of the Study

Heterogeneity in study designs (RCTs vs. observational), follow-up durations, and variable bleeding definitions may limit generalizability and complicate safety comparisons. Although subgroup analyses were prespecified, they were underpowered to detect slight differences. In addition, publication bias could have favored positive results, though Egger’s test (p = 0.525) and funnel plot inspection suggested minimal influence.

Future Directions

Future research should aim to standardize bleeding endpoints to improve cross-trial comparability, investigate biomarkers (e.g., platelet reactivity, genetic testing) to identify patients most likely to benefit from monotherapy versus dual therapy, and assess the cost-effectiveness of newer regimens (e.g., ticagrelor monotherapy) in real-world practice. Pragmatic, risk-stratified clinical trials are warranted.

## Conclusions

Aspirin endures as a fundamental, cost-effective agent for secondary MI prevention, demonstrating a consistent 19% reduction in recurrent cardiovascular events. However, the era of a universal, one-size-fits-all aspirin regimen is obsolete. Optimal patient outcomes now hinge on a personalized strategy that carefully balances ischemic protection against iatrogenic bleeding risk. For most patients, this entails defaulting to a lower-dose aspirin regimen (75-100 mg daily), which provides an optimal efficacy-safety profile by maximizing anti-ischemic effects while minimizing gastrointestinal toxicity. In clinical scenarios where bleeding risk is paramount, such as in patients with a history of GI bleeding, concomitant anticoagulant use, or high frailty, alternative monotherapy with a P2Y₁₂ inhibitor (e.g., clopidogrel) presents a validated and guideline-endorsed strategy that foregoes aspirin without sacrificing ischemic protection. Conversely, intensification of therapy remains a critical tool for a distinct subset of high-risk patients. For those with a compelling ischemic profile, such as individuals with diabetes, multivessel disease, or a complex recent PCI, extended-duration DAPT provides a significant reduction in thrombotic events, albeit at the cost of a quantifiable increase in bleeding. This calculated trade-off underscores the clinician's essential role in risk stratification. The future of secondary prevention lies in this nuanced, patient-centric paradigm, moving beyond uniform protocols to embrace tailored treatment algorithms. Further refinement of these strategies would depend on integrating emerging tools, such as genetic testing, platelet function assays, and validated risk scores, to precisely calibrate therapy and maximize the net clinical benefit for each individual.
